# Tolerability of inhaled N-chlorotaurine in an acute pig streptococcal lower airway inflammation model

**DOI:** 10.1186/1471-2334-11-231

**Published:** 2011-08-29

**Authors:** Martin Schwienbacher, Benedikt Treml, Anna Pinna, Ralf Geiger, Hannes Reinstadler, Iris Pircher, Elisabeth Schmidl, Christian Willomitzer, Johannes Neumeister, Michael Pilch, Maria Hauer, Thomas Hager, Consolato Sergi, Sabine Scholl-Bürgi, Thomas Giese, Alexander Löckinger, Markus Nagl

**Affiliations:** 1 Department of Pediatrics, Division of Cardiology, Pulmology, Allergology and Cystic Fibrosis, and Division of Neonatology, Neuropediatrics and Inborn errors of metabolism, Innsbruck Medical University, Innsbruck, Austria; 2 Department of Anaesthesiology and Critical Care Medicine, Innsbruck Medical University, Innsbruck, Austria; 3 Department of Hygiene, Microbiology and Social Medicine, Division of Hygiene and Medical Microbiology, Innsbruck Medical University, Innsbruck, Austria; 4 Institute of Pathology, Innsbruck Medical University, Innsbruck, Austria, and Department of Laboratory Medicine and Pathology, University of Alberta, Edmonton, AB, Canada; 5 Department of Immunology, University of Heidelberg, Heidelberg, Germany

## Abstract

**Background:**

Inhalation of N-chlorotaurine (NCT), an endogenous new broad spectrum non-antibiotic anti-infective, has been shown to be very well tolerated in the pig model recently. In the present study, inhaled NCT was tested for tolerability and efficacy in the infected bronchopulmonary system using the same model.

**Methods:**

Anesthetized pigs were inoculated with 20 ml of a solution containing approximately 10^8 ^CFU/ml *Streptococcus pyogenes *strain d68 via a duodenal tube placed through the tracheal tube down to the carina. Two hours later, 5 ml of 1% NCT aqueous solution (test group, n = 15) or 5 ml of 0.9% NaCl (control group, n = 16) was inhaled via the tracheal tube connected to a nebulizer. Inhalation was repeated every hour, four times in total. Lung function and haemodynamics were monitored. Bronchoalveolar lavage samples were removed for determination of colony forming units (CFU), and lung samples for histology.

**Results:**

Arterial pressure of oxygen (PaO_2_) decreased rapidly after instillation of the bacteria in all animals and showed only a slight further decrease at the end of the experiment without a difference between both groups. Pulmonary artery pressure increased to a peak 1-1.5 h after application of the bacteria, decreased in the following hour and remained constant during treatment, again similarly in both groups. Histology demonstrated granulocytic infiltration in the central parts of the lung, while this was absent in the periphery. Expression of TNF-alpha, IL-8, and haemoxygenase-1 in lung biopsies was similar in both groups. CFU counts in bronchoalveolar lavage came to 170 (10; 1388) CFU/ml (median and 25 and 75 percentiles) for the NCT treated pigs, and to 250 (10; 5.5 × 10^5^) CFU/ml for NaCl treated pigs (p = 0.4159).

**Conclusions:**

Inhaled NCT at a concentration of 1% proved to be very well tolerated also in the infected bronchopulmonary system. This study confirms the tolerability in this delicate body region, which has been proven in healthy pigs previously. Regarding efficacy, no conclusions can be drawn, mainly because of the limited test period of the model.

## Background

N-chlorotaurine is an endogenous mild oxidant formed from hypochlorous acid and taurine during the oxidative burst of granulocytes and monocytes [1,2]. It can be synthesized chemically as sodium salt (Cl-HN-CH_2_-CH_2_-SO_3_-Na, NCT) [3] and has been applied successfully as a new broad spectrum non-antibiotic anti-infective in clinical studies to different body sites [4], for instance the eye [5], the outer ear [6], and skin ulcerations [7]. Because of its high tolerability, also susceptible regions come into question for topical therapy. Recently, inhaled NCT at a concentration of 1% (w/v) in water was very well tolerated in a pig model [8]. Compared to 0.9% sodium chloride (w/v), no worsening of oxygenation parameters (e.g. arterial pressure of oxygen, alveolo-arterial difference of oxygen partial pressure) and pulmonary artery pressure could be found. There were no differences in histology of lung samples between 1% NCT and 0.9% NaCl, and the surfactant function was not impaired by 1% NCT [8]. A five times higher NCT concentration (5%) led to significantly higher pulmonary artery pressure, but to no further measureable disorders.

Based on these findings, in the present study we wanted to investigate the effects of inhaled 1% NCT to the bronchopulmonary system of pigs whose airways had previously been infected with *Streptococcus pyogenes*. In addition, we attempted to test the efficacy of NCT, being aware of the limited observation time possible in this pig model.

## Methods

### Test solutions

Pure NCT as a crystalline sodium salt (molecular weight 181.57) was prepared according to [3]. Purity was proved by spectrophotometry [3]. NCT was dissolved in sterile and pyrogen-free distilled water to solutions with a final concentration of 1% (w/v; 55 mM). Solutions were sterile-filtered and stored at 2-4°C for maximally one week. Sterile and pyrogen-free 0.9% sodium chloride (B. Braun Melsungen AG, Melsungen, Germany) was used as a control. To warrant double-blinding, test and control solutions were filled in similar flasks. All flasks were numbered consecutively in accordance with the randomization code.

### Animals

The experiment was approved by the Austrian Federal Animal Investigational Committee, and animals were managed in accordance with the National Institutes of Health guidelines http://grants.nih.gov/grants/olaw/olaw.htm. Healthy, 4-week-old white farm pigs were used in the study. They were anesthetized during the whole experiment.

### Bacteria

*Streptococcus pyogenes *d68, kindly provided by J. Hildebrandt, Sandoz Scientific Center Vienna [9], deep frozen for storage was grown on Columbia agar with 5% (v/v) sheep blood. The strain was chosen because of its high virulence proven in a mouse model [9], since we aimed to cause rapid inflammation due to the limited test period of the pig model. Single colonies from the agar plates were grown at 37°C for 14-15 h in 20 ml tryptic soy broth (Merck) without agitation. Subsequently, the suspension was centrifuged for 10 min at 1800 × g, and the bacteria were washed twice with 0.9% NaCl before application. Colony forming units (CFU) counts were determined by spreading 50 μL aliquots of adequate dilutions in duplicate onto blood agar with an automated spiral plater (model WASP 2, Don Whitley Scientific Limited, Shipley, UK). The plates were incubated at 37°C, and the CFU were counted after 24 and 48 h.

### Study design and treatment

The pigs were randomly assigned to the test group (4 inhalations with 5 ml 1% NCT) or to the control group (4 inhalations with 5 ml 0.9% NaCl). Sixteen animals per group were included. One animal of the NCT group demonstrated a very bad lung and circulation function after anaesthesia and was excluded from the study so that finally 15 pigs received full treatment and were evaluated in the test group compared to 16 in the control group. Maximally two pigs were treated and evaluated on the same day. Application of test solutions and measurements of evaluation parameters were performed in a blinded way. Thirty min after anesthesia, baseline parameters were recorded (criteria for baseline: arterial pH between 7.35 and 7.40, pCO_2 _between 35 and 40 mmHg, pO_2 _at least 80 mmHg). Then, a solution of 20 ml containing 0.5 - 5.0 × 10^8 ^CFU/ml *S. pyogenes *d68 with no difference between the test and control group was instilled via the endotracheal tube (ID 6,0 Lo-Contour Murphy, Mallinckrodt, Ireland).

120 min later, the first inhalation was performed, followed by three further inhalations at intervals of 60 min with 5 ml of 1% NCT or 0.9% NaCl. Approximately thirty min after the last inhalation, the animals were euthanized, a bronchoalveolar lavage (BAL) with 10 ml of 0.9% NaCl in each lung was performed, and lung samples were removed. Parameters of oxygenation and circulation were monitored. Baseline values and values every 30 min after the first inhalation were recorded. Blood samples were taken every 30 min, arterial and expiratory multiple inert gas elimination technique was performed at baseline, before first inhalation, 30 min, 120 min and 240 min after the first inhalation. Blood samples for quantifying serum taurine and chloride levels were taken at baseline and at the end of the experiment.

### Anaesthesia of pigs

Anaesthesia was induced with ketamine (25 mg/kg IM) and azaperone (2 mg/kg IM), followed by intravenous propofol (1-2 mg/kg IV). After the trachea had been intubated, piritramide 15 mg was administered. Anaesthesia was then maintained with propofol (10 - 15 mg kg^-1 ^h^-1^) and piritramide boluses (7.5 mg each, summary dosis of 1.65 mg/kg). Ringers solution (14 ml kg^-1 ^h^-1^), 0.9% NaCl (20 ml kg^-1 ^h^-1^) and a 4% gelatine solution (6 ml kg^-1 ^h^-1^) were administered throughout the procedure. Lungs were ventilated in a volume controlled mode (Evita 2, Dräger Medical, Vienna, Austria) at an inspiratory fraction of oxygen (FIO_2_) of 0.21 and a tidal volume (VT) of 10 - 12 ml/kg at 15 breaths/min, positive end expiratory pressure set at 5 cm H_2_O. Tidal volume was then adjusted to achieve an arterial pressure of carbon dioxide (PaCO_2_) between 35 and 40 mmHg, resulting in an average min volume of 180 ml kg^-1 ^min^-1^. A standard lead II ECG was used to monitor cardiac rhythm. Body temperature was maintained between 38°C and 39°C using an electric heating blanket.

### Animal instrumentation

Two venous catheters were inserted percutaneously into auricular veins for inert gas infusion and continuous infusion of propofol. A no. 7F thermistor-tipped Swan-Ganz catheter (Baxter Edwards, Irvine, CA) was inserted into the right jugular vein and advanced into a main pulmonary artery by use of direct-pressure monitoring. This allowed measurement of cardiac output, mean pulmonary arterial pressure (PAPM), pulmonary artery occlusion pressure (PCWP) and mixed venous blood sampling. The left femoral artery was cannulated with a no. 6F Avanti+ introducer (length 11 cm, Cordis Corporation, Miami, USA) for measurement of systemic pressure and arterial blood sampling. The urine volume was measured with a balloon catheter (Rüsch Brillant Silicone Catheter, Teleflex Medical, Kamunting, Malaysia).

### Haemodynamic measurements

Systolic, diastolic and mean arterial and pulmonary arterial pressure (SAP, DAP, MAP; PAP, sPAP, dPAP, mPAP), central venous pressure (CVP) and pulmonary artery occlusion pressure (PCWP) were measured with an ICU monitor (Datex AS/3, Sanitas, Salzburg, Austria) with standard pressure transducers (single transducer set no. REF 686685, model PM SET 1DT-XX 1 Rose, Becton Dickinson Critical Care Systems, Singapore) that had been zeroed to the level of the right atrium. Cardiac output was measured with an Inline Injektat Sensor (set no. REF 1900210, Angiokard Medizintechnik GmbH + CoKg, Friedeburg, Germany) and a no. 7 Swan-Ganz catheter (Edwards Lifesciences Austria GmbH, Vienna, Austria). The mean of 3 determinations of cardiac output was recorded.

### Blood Gas and Metabolic Measurements

Arterial and mixed venous samples (2 ml each) were collected simultaneously and immediately analyzed for oxygen and carbon dioxid partial pressure, pH, haemoglobin concentration, haematocrit, and electrolytes with a Bayer Rapidlab 865 blood gas analyzer (Bayer Healthcare, Fernwald, Germany).

### Respiratory Measurements

Airway pressures, expiratory tidal volume, expiratory min volume, and respiratory rate values were recorded by the built-in detectors of the ventilator. Ventilation-perfusion distributions were determined using the multiple inert gas elimination technique (MIGET, 5890 Series II Gas Chromatograph, Hewlett Packard Company, Wilmington, USA [10,11]).

### Inoculation and NCT treatment protocol

After instrumentation of the animals and a stabilization period of 30 min, baseline measurements (haemodynamics, ventilation parameters, blood gases, MIGET) were performed. Then, the suspension of 20 ml containing *S. pyogenes *d68 was instilled via the endotracheal tube. Two hours later we performed the first inhalation with an ultrasonic nebulizer (Optineb^®^-K Mobile Ultrasonic Nebulizer ON-100/5 2,4 MHz from NEBU-TEC med. Produkte, Elsenfeld, Germany). The Mass Median Aerodynamic Diameter of the particles produced by this nebulizer was 2.3 μm, which warranted sufficient penetration of the NCT-containing aerosol to the bronchioli and alveoli. Delivery of the aerosol was provided during the inspiration phase by direct connection to the inspiratory side of the Y-piece. At the end of the experimental runs, animals were killed using potassium chloride in conjunction with a deepening of the general anaesthesia.

### Quantification of *S. pyogenes *in bronchoalveolar fluid

Immediately after euthanizing the animals, the chest was opened and a peripheral bronchus was irrigated with 10 ml of 0.9% NaCl. Undiluted bronchial fluid as well as aliquots of this fluid tenfold diluted in 0.9% NaCl (50 μl each) were spread in duplicate on blood agar plates with the automatic spiral plater, and CFU were counted after incubation at 37°C for 24 h and 48 h. *S. pyogenes *was differentiated by the mucous colony form from other bacteria. Other bacterial species growing on the plates were counted separately. All bacterial species were identified biochemically (Vitek^®^, BioMerieux, Mary l'Etoile, France).

### Pharmacokinetics of NCT

To address the local pharmacokinetics in the lung, 5 ml of the bronchoalveolar fluid (see previous paragraph) were removed, potassium iodide was added in excess, and the formed coloured tri-iodide was measured spectrophotometrically at 350 nm, its maximum wavelength (absorption coefficient 22900 mol^-1 ^cm^-1 ^[12]).

To address systemic uptake of NCT, the taurine concentration in the serum was determined at the baseline and after the last inhalation in 5 samples of the test and 6 samples of the control group. If NCT in amounts used for inhalation comes into contact with blood, it is immediately inactivated by thio groups and degrades into taurine and chloride. Therefore, taurine is an ideal measure for systemic uptake of topically applied NCT. Taurine was analyzed by ion-exchange chromatography with ninhydrin detection (AminoTac, JLC-500/V, Jeol) [13,14]. The disulfide cystine as an oxidation product of cysteine and methionine were evaluated, too.

### Histology

Subsequent to euthanizing the animal, artificial ventilation was continued up to removal of lung samples of the upper and lower lobes taken from both a central and a peripheral area. Lung tissue was subjected to histological processing according to standard procedures, i.e. formalin fixation (buffered 4% formalin, v/v) and paraffin-embedding.

Moreover, fresh tissue was used to extract protein, DNA and RNA according to commercial kits instructions. The tissue was routinely stained with haematoxylin and eosin, periodic acid Schiff (glycogen staining), elastica van Gieson (elastic fibers of the intrapulmonary blood vessels), and reticulin (reticulin fibers). Haematoxylin-eosin staining was used to evaluate the infiltration with neutrophilic granulocytes, eosinophilic granulocytes, lymphocytes, erythrocytes, atelectasis, bronchiolitis, bronchitis, lymphangiectasia, and capillary congestion. PAS staining was used to evaluate the variability of the amount of goblet cells, mucus, detachment of epithelial cells and epithelial changes. Elastica van Gieson staining was used to detect the fragmentation of the external and internal laminae of the blood vessels. Reticulin staining was used to detect collapse of fibers, thinning or thickening of the alveolar septa, the presence of emphysema, bullae and thickening of the pleura.

### In situ determination of cytokines

Expression of interleukin (IL)-8, tumor necrosis factor alpha (TNF-alpha), and haemoxygenase 1 (HO-1) was investigated by mRNA analysis according to [15]. Real-time RT-PCR was performed as described in detail in our previous study on biopsies collected in RNAlater (Ambion, Austin, TX, USA) and stored at minus 80°C until analysis [8]. Briefly, tissue was disrupted by 3 to 4 runs with the RiboLyser (ThermoHYBAID, Heidelberg, Germany) in lysing matrix „D" tubes (Q-BIOgen, Heidelberg) containing 400 μl lysis buffer from the MagnaPure mRNA Isolation Kit II (ROCHE Diagnostics, Mannheim, Germany). Lysate was mixed with capture buffer containing oligo-dT, centrifuged, and transferred into a MagnaPure sample cartridge. mRNA was isolated with the MagnaPure-LC device using the mRNA-II standard protocol.

An aliquot of 8.2 μl mRNA was reversely transcribed using AMV-RT and oligo- (dT) as primer (First Strand cDNA synthesis kit, Roche) according to the manufactures protocol in a thermocycler. cDNA samples were stored at -20°C until PCR analysis. Primer sets for pig GAPDH, IL-8, HO-1 and TNF-alpha optimized for the LightCycler^® ^(RAS, Mannheim, Germany) were developed and purchased from SEARCH-LC GmbH, Heidelberg. The PCR was performed with the LightCycler^® ^FastStart DNA Sybr GreenI kit (RAS) according to the protocol provided in the parameter specific kits with evaluation specifications as published previously [8]. To correct for differences in the content of mRNA, the calculated copy numbers were normalized according to the average expression of the housekeeping gene GAPDH. Values were thus given as input adjusted copy number per μl of cDNA.

### Statistical Analysis

Shapiro-Wilk normality test was applied to test for Gaussian distribution of the data. Accordingly, they are presented as mean values and standard deviations or standard errors of the mean or as median and percentiles. For hemodynamics, blood gas, respiratory measurements, pharmacokinetic parameters, and HO-1, Student's unpaired *t *test or one-way ANOVA and Bonferroni's multiple comparison test was used to test for a difference between the test and control group, while the paired *t *test was applied to test for differences between different time-points in each group. Mann-Whitney U test was used to compare CFU counts of bacteria in bronchoalveolar fluid and TNF-alpha and IL-8 in lung biopsies. Chi-square and Fisher's test were used for evaluation of changes found in histology. Student's *t *test for unpaired samples was applied for comparison of the number of granulocytes counted by microscopy in lung histology sections. Calculations were done with GraphPad Prism 5 software. P < 0.05 was considered significant for all tests.

## Results

### Pulmonary gas exchange

**Figure 1:**
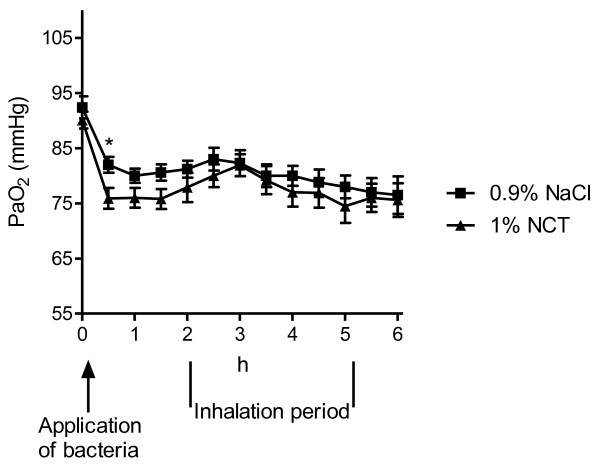
**Arterial partial pressure of oxygen (PaO_2_) of the pigs during the inhalation period**. Mean values ± SEM of 15 (NCT) and 16 (NaCl) animals. * p < 0.05 between NCT and NaCl; p > 0.05 between NCT and NaCl for all other time-points (unpaired t test); p < 0.01 between time zero and all other time-points (paired t test).

Arterial partial pressure of oxygen (PaO_2_) values at the beginning of anaesthesia were within normal range in all animals without any intergroup difference (90.1 ± 5.9 mmHg and 92.4 ± 7.8 mmHg, test versus control animals), reflecting healthy, anesthetized and ventilated animals. After application of streptococci via the respiration tube, PaO_2 _decreased rapidly within 30 min to 75.9 ± 7.3 and 82 ± 5.6 mmHg, respectively (Figure 1, p < 0.01 versus baseline for both groups). At the beginning of the inhalations, the values were 77.9 ± 10.3 and 81.2 ± 6.0 mmHg. PaO_2 _remained constant over the inhalation period in all animals and was 75.6 ± 11.8 and 76.5 ± 13.2 mmHg, respectively, 4 hours later (p > 0.1 between groups and over that time).

Alveolo-arterial difference of oxygen partial pressure (AaDO_2_) increased from 22.2 ± 13.3 mmHg (17.8 ± 8.6 for controls) at the baseline to 32.5 ± 9.4 mmHg (31.2 ± 7.0) at the beginning of the inhalations, and to 39.8 ± 12.5 mmHg (41.2 ± 11.5) at the end of the experiment (p > 0.1 between NCT and NaCl; p < 0.05 between these time points in each group). Arterial oxygen saturation remained constant between 94% and 97% in all animals. A slight increase of the pH from about 7.47 to 7.51 (p < 0.05 for the change over time from the beginning to the end of the inhalations, but p > 0.05 between groups) and an according slight decrease of the arterial carbon dioxide pressure from about 36.2 to 32.5 mmHg was monitored. Ventilation-perfusion distributions determined with MIGET showed no differences between the test (normal VA/Q of Q ratio after 4 hours 96% for 1% NCT) and control groups (97%).

### Haemodynamics

**Figure 2:**
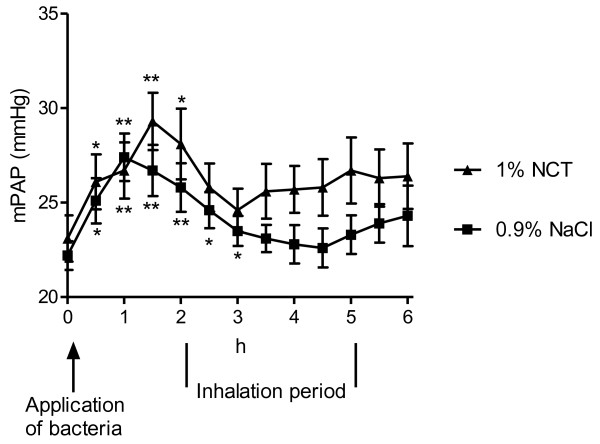
**Mean pulmonary artery pressure (mPAP) of the pigs during the inhalation period**. Mean values ± SEM of 15 (NCT) and 16 (NaCl) animals. P > 0.05 between NaCl and 1% NCT for each time-point (unpaired t test); * p < 0.05 versus baseline (paired t test); ** p < 0.01 versus baseline (paired t test).

Pulmonary artery pressure increased during the first 1.5 h after challenge with bacteria (p < 0.01), decreased again in the following hour and remained constant during the inhalation period (Figure 2). It seemed to decrease again to lower values in the control than in the test group, but the difference was not significant (p = 0.077 and 0.095 for 4 and 4.5 h, respectively, and p > 0.1 for all other time-points).

No differences between the test and control group were found in further haemodynamics (p > 0.05 for all parameters). Heart rate increased from 81 ± 16/min (test group) and 76 ± 11/min (control group) at the baseline to 92 ± 17 and 86 ± 11 at the beginning of the inhalations 2 h later (p < 0.01), and to 104 ± 20/min in both groups after 6 h (p < 0.05). Systolic blood pressure increased from 120 ± 17 and 115 ± 12 at the baseline to 147 ± 23 and 147 ± 20, respectively, after 2 h (p < 0.01) and decreased again slowly during the inhalation period (136 ± 12 and 140 ± 14 after 6 h; p = 0.03 and 0.06, respectively). Diastolic blood pressure behaved in a similar way, i.e. increased from 70 ± 15 and 68 ± 12 to 99 ± 20 and 101 ± 15 after 2 h (p < 0.01 for both), and came to 90 ± 16 (p < 0.05) and 94 ± 17 (p > 0.05) after 6 h. No notable changes were found in central venous pressure and pulmonary capillary wedge pressure.

### Microbiology

**Figure 3:**
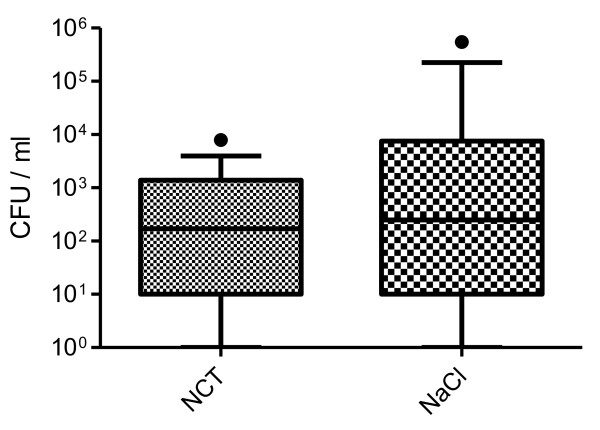
**Counts of CFU of *S. pyogenes *d68 in bronchoalveolar fluid after 4 inhalations**. Median and 10-90 percentiles of 15 (NCT) and 16 (NaCl) pigs; p = 0.4159.

From BAL performed immediately after euthanasia and opening of the chest, *S. pyogenes *d68 could be cultivated in 13 of 15 animals in the NCT group and in 12 of 16 animals in the control group. The median and percentiles of *S. pyogenes *CFU found in quantitative cultures are depicted in Figure 3. The median (25; 75 percentile) was 170 (10; 1388) CFU/ml for the NCT treated pigs with a range of 0 to 7900, and 250 (10; 7500) CFU/ml for NaCl treated pigs with a range of 0 to 12500 and one outlier of 5.5 × 10^5^. There was no difference between both groups with p = 0.4159 (Mann-Whitney U test).

In addition, the total bacterial count of all species that were found on the plates was determined. Additional species were mainly *Pseudomonas aeruginosa*, *Streptococcus suis*, and *Staphylococcus aureus*, which were not instilled via the respiratory tube. The median (25; 75 percentile) was 1710 (276; 7284) CFU/ml for the NCT treated pigs with a range of 150 to 22300, and 3000 (710; 10350) CFU/ml for NaCl treated pigs with a range of 50 to 5.5 × 10^5^. There was again no difference between both groups with p = 0.2599.

### Pharmacokinetics

In bronchoalveolar fluid, no oxidative capacity could be found approximately 30 min after the last inhalation.

Taurine levels in serum were 55.0 ± 27.1 μmol/l for NCT and 52.3 ± 22.4 μmol/l for NaCl (p > 0.1) at time zero, and 45.5 ± 25.2 μmol/l for NCT and 45.8 ± 20.1 μmol/l for NaCl (p > 0.1) after the last inhalation (mean values ± SD; n = 5 for NCT and n = 6 for NaCl). Cystine levels came to 10.2 ± 9.1 μmol/l for NCT and 7.9 ± 4.4 μmol/l for NaCl (p > 0.1) at time zero, and 17.0 ± 7.9 μmol/l for NCT and 13.4 ± 3.7 μmol/l for NaCl (p > 0.1) after the last inhalation. The values for methionine were 19.3 ± 4.0 μmol/l for NCT and 16.6 ± 5.1 μmol/l for NaCl (p > 0.1) at time zero, and 23.2 ± 7.0 μmol/l for NCT and 26.3 ± 8.7 μmol/l for NaCl after the last inhalation (p > 0.1). The increase of cystine levels after the last inhalation was significant for both the NCT (p < 0.011) and the NaCl group (p = 0.0001), whereas there were no differences between the groups.

### Histology

In the histological sections, a mild bronchiolitis and bronchitis was detectable. It was markedly present in the carina area and moderate in the area distant to carina. In the peripheral area, the inflammation was less significant. In the upper lung lobes, no inflammation was found.

**Figure 4:**
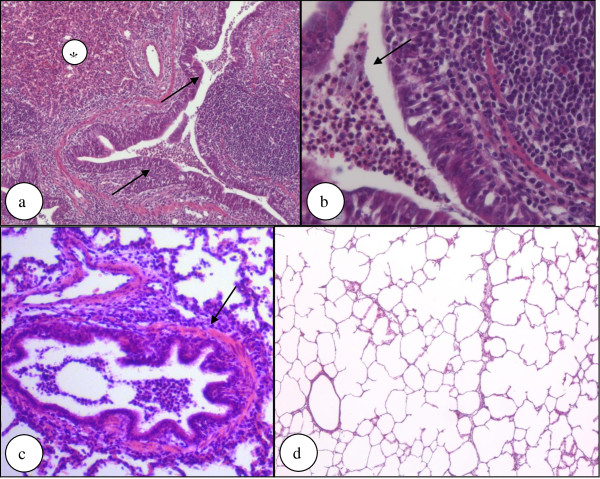
**Histological evaluation of lung samples**. Figure 4a-b show lung tissue from a pig treated with NCT showing atelectases (*) and infiltration of the bronchial and alveolar system by neutrophilic granulocytes that are present in the lumen of a bronchial airway (arrows) (hematoxylin-eosin staining; original magnification 100× (a) and 400 × (b); p > 0.1 for frequency of occurrence between test and control group). Figure 4c shows a mild increase of thickness of the bronchial wall on serial sections, although this finding was patchy in the same lung and not uniform in the same group (arrow) (hematoxylin-eosin staining; original magnification 200×). Figure 4d shows lung tissue from a pig treated with NaCl showing neither atelectasis nor neutrophilic granulocytic infiltration (hematoxylin-eosin staining, original magnification 50x; p > 0.1 for frequency of occurrence between test and control group).

Examination of the different lung fields using a random number generator and ImageJ as well as manual counting mostly identified two different histologic patterns. In the first one, there were lung fields that showed no inflammation or many clear areas with leukocytic infiltration marginalized to some bronchial lumina and very few leukocytes in single alveoli (Figure 4a-b). This pattern was present in 12 out of 15 pigs in the NCT group and in 13 out of 16 pigs in the control group (p > 0.1). In the second pattern, leukocytic infiltration was diffuse involving both the bronchial and alveolar systems, represented in 3 pigs of the NCT and in 2 pigs of the control group (p > 0.1). Both patterns showed a mild increase of the thickness of the bronchial wall (Figure 4c). However, this finding was patchy in the same lung and not uniform between the animals (p > 0.1 between both groups). No inflammation at all could be found in few specimens (1 in the NCT, 3 in the NaCl group, p > 0.1) (Figure 4d), whilst a few cases showed mostly clear areas with focal infiltration with polymorphonuclear leukocytes in bronchial lumina (8 in the test, 5 in the control animals, p > 0.1). Moreover, there were multinucleated giant cells in a single case in each group. Plasmacellular infiltration was found in 1 pig of the NCT group, and macrophage involvement in 5 of the test and in 2 of the control pigs (p > 0.1). Granulocytes were counted in three microscopic fields of at least two samples per animals at a 400-fold magnitude, and the numbers came to 51.1 ± 68.5 per field (mean value ± SD; range 0 - 321) for test samples and to 35.2 ± 64.8 per field (mean value ± SD, range 0 - 345) for controls (p > 0.1).

### In situ determination of cytokines

Biopsies near the carina from 10 animals of the NCT group and 8 animals from the control group were randomly selected and analyzed. The adjusted transcript values (number/μl cDNA) are listed in Table 1. The expression of the investigated genes was less variable than in the tolerability study [8], but the absolute values were higher. There were no differences between the test and control group (p > 0.6).

**Table 1:** Gene expression by qRT-PCR in lung samples after inhalation, adjusted mRNA transcript values (number/μl cDNA)

	1% NCT	0.9% NaCl
HO-1^a^	7458	7450
	(2823)	(4673)
TNF- alpha^b^	1291	1101
	(767; 2568)	(590; 4600)
IL-8^b^	1859	1188
	(933; 20214)	(771; 6695)

^a ^mean values and (SD)

^b ^median and (25%; 75% percentiles)

p > 0.6 between NCT and NaCl for all values.

## Discussion

Recently, the pig model proved to be very suitable and sensitive to test the tolerability of inhaled NCT [8]. In the present study, we tested if the concentration of 1% NCT, which was well tolerated in the healthy pig, was also tolerable in the infected lung.

Oxygenation (e.g. pressure of oxygen) and hemodynamic (e.g. pulmonary artery pressure) measures worsened quickly in the present study after bronchial application of the bacteria, but they remained either constant during the inhalation period or worsened moderately without a difference between the test and control group. This course indicates that *S. pyogenes *had a rapid impact on the lung function in the first 1-2 hours, i.e. before the inhalations were started. Previous studies using similar pig models are in compliance with this finding, since a rapid decrease of PaO_2 _was detected after bronchial application of *P. aeruginosa *[16,17] and Group B streptococci [18]. Increased respiratory rate was seen in pigs challenged with *Actinobacillus pleuropneumoniae *[19,20]. The absence of a marked worsening in the following time in our study suggests that the bacteria did not cause a massive, life-threatening inflammation in the first 6 hours after the challenge, similar to the just mentioned previous studies [16-20]. This is supported by the histological findings of partial leukocyte infiltration in the central parts of the lung, which indeed had been observed in a similar percentage of 60 - 70% of the pigs in the previous tolerability study in healthy pigs, too [8]. Therefore, it remains unclear to what extent the bacteria contributed to the histologically visible inflammation. In any case, their influence on the clinical parameters was obvious. Moreover, transcript values of IL-8, TNF-alpha, and HO-1 were equal in the NCT and NaCl group, but higher than in the previous pig inhalation study, where no bacteria were applied [8]. This clearly indicates that these elevated levels were caused by the streptococci and not by NCT.

Since both the course and absolute values of all evaluated blood gas, haemodynamic, and histological parameters were similar in the test and control group, inhaled 1% NCT proved to be well tolerated in the infected pig lung, too. The mean pulmonary artery pressure values appeared to be a little higher in the test group (Figure 2). However, this difference did not reach statistical significance at any time point. Moreover, the pressure increased to higher levels in the test than in the control group already after instillation of the bacteria and *before *the beginning of the inhalations. Similar to the controls, it decreased after the first inhalation, and approximated again to the value of the controls after 4 inhalations. Because of all these reasons, we estimate these findings as no difference in pulmonary artery pressure between test and control group. Furthermore, this conclusion is in agreement with the previous study in healthy pigs [8].

It is true that the observation period of six hours after application of the bacteria was limited, but prolongation of the anaesthesia of the pigs for a few days as in few previous studies [16,17] has not been possible in our unit. While this - in all likelihood - did not impact the testing of the tolerability of NCT, a reliable evaluation of its efficacy turned out to be impossible within that observation period. Actually, development of pneumonia with severe clinical signs and increase of markers of inflammation in the blood needed at least 12-24 h after bronchial inoculation of *P. aeruginosa *[16,17] or inhalation of nebulized *A. pleuropneumoniae *[19,20]. We tried to overcome this problem in part using *S. pyogenes *d68, a strain highly virulent at least in the mouse [9], but we were not successful to induce a massive inflammation within short time. The high range of CFU counts in BAL indicates a randomly scattered distribution of bacteria in the bronchial system, which fitted well to the different histological findings in samples from different areas. Application of bacteria in a nebulized form may lead to a more equal distribution, but this was not possible with a highly virulent strain. As an approach to test this, we applied *Micrococcus luteus *(4-5 ml of 3-4 × 10^8 ^CFU/ml) through the nebulizer in four additional pigs, but not more than 10 CFU/ml could be grown, and 5 of 8 BAL cultures were negative.

The absence of oxidative activity in the bronchoalveolar fluid approximately 30 min after inhalation of 5 ml 1% NCT confirms the results in healthy pigs [8] and indicates a short half-life of NCT in the lung. Taking into account the large surface of the lung, the limited amount of 4 × 5 ml inhaled and the rapid decay of NCT into taurine and chloride in the presence of organic substances [21], this result fits very well to previous knowledge. The short half-life together with the mild activity of NCT explain also the absence of a difference in the CFU counts between the control and test group indicating the absence of a rapid massive killing of bacteria by NCT. Further reasons may be the scattered distribution of bacteria (see above) and also of NCT. One has to be aware that particularly in the inflamed bronchopulmonary system with the occurrence of atelectases, distribution of inhaled substances is never completely homogenous. However, these considerations do not exclude a therapeutic efficacy. NCT is known to impact the virulence of pathogens after very short sublethal contact times of 1 min and to exert a postantibiotic effect [9,22,23], and it has shown efficacy in conjunctivitis in the human eye despite loss of oxidative activity within a few min [24,25].

## Conclusions

N-chlorotaurine was well tolerated at a concentration of 1% upon inhalation in an acute pig streptococcal lower airway inflammation model. This study confirms the tolerability in this delicate body region, which has been proven in healthy pigs previously. To evaluate the efficacy of NCT, the model turned out to be not suitable, mainly because of the limited test period.

## Competing interests

MN has applied for intellectual property rights protection on the inhalative application of NCT and started a cooperation with a pharmaceutical company that develops NCT and related chloramines as antiinfectives. All other authors declare that they have no competing interests.

## Authors' contributions

All authors read, performed according corrections, and approved the final manuscript. Additionally, the authors' tasks were specifically the following. MS and BT planned, performed and guided the animal work, did evaluation and statistics, and wrote the animal model. AP performed animal and microbial work, sample preparation and collection, data management and evaluation. RG did planning of the study, establishment of the inhalation devices, and evaluation. HR, IP, ES, CW, JN, MP, and MH were involved in the performance of the animal model including the inhalations, sample collection, and data management. TH performed and evaluated histology. CS guided, performed and evaluated histology, and wrote the histological part of the manuscript. SSB guided and evaluated the pharmacokinetic tests. TG performed in situ determination of cytokines and evaluation. AL planned the study, guided and coordinated the animal tests. MN planned the study, coordinated all involved scientists and of their work, guided the microbial work, performed evaluation including statistics, and wrote the manuscript.

## Pre-publication history

The pre-publication history for this paper can be accessed here:

http://www.biomedcentral.com/1471-2334/11/231/prepub
